# Multiferroic Cantilevers Containing a Magnetoactive Elastomer: Magnetoelectric Response to Low-Frequency Magnetic Fields of Triangular and Sinusoidal Waveform

**DOI:** 10.3390/s22103791

**Published:** 2022-05-17

**Authors:** Gašper Glavan, Inna A. Belyaeva, Mikhail Shamonin

**Affiliations:** East Bavarian Centre for Intelligent Materials (EBACIM), Ostbayerische Technische Hochschule (OTH) Regensburg, Seybothstr. 2, D-93053 Regensburg, Germany; inna.belyaeva@oth-regensburg.de

**Keywords:** magnetoactive elastomer, piezoelectric polymer, multilayer cantilever, direct magnetoelectric effect, magnetic field sensing

## Abstract

In this work, multiferroic cantilevers comprise a layer of a magnetoactive elastomer (MAE) and a commercially available piezoelectric polymer-based vibration sensor. The structures are fixed at one end in the horizontal plane and the magnetic field is applied vertically. First, the magnetoelectric (ME) response to uniform, triangle-wave magnetic fields with five different slew rates is investigated experimentally. Time and field dependences of the generated voltage, electric charge, and observed mechanical deflection are obtained and compared for four different thicknesses of the MAE layer. The ME responses to triangular and sinusoidal wave excitations are examined in contrast. Second, the ME response at low frequencies (≤3 Hz) is studied by the standard method of harmonic magnetic field modulation. The highest ME coupling coefficient is observed in the bias magnetic field strength of ≈73 kA/m and it is estimated to be about 3.3 ns/m (ME voltage coefficient ≈ 25 V/A) at theoretically vanishing modulation frequency (f→0 Hz). Presented results demonstrate that the investigated heterostructures are promising for applications as magnetic-field sensors and energy harvesting devices.

## 1. Introduction

The direct magnetoelectric (ME) effect is the induced change in the electric polarization *P* due to a change in the applied magnetic field strength *H* [1,2]. Natural, single-phase materials demonstrating the direct ME effect are rather rare and the ME coupling is very low (ME voltage coefficient $\alpha_{V}^{H}\approx 0.1-10$ mV/A) [3]. These materials are usually designated as multiferroic, which means that at least two types of spontaneous ferroic orders, such as ferroelectricity and ferromagnetism, are present [3,4]. The ME effect can be significantly increased in the composite (two-phase) materials, which exhibit much higher ME coupling [1,5]. Most conventional multiferroic composite materials are layered heterostructures made from rigid materials (Young’s modulus $Y∼10^{11}$ Pa), such as piezoelectric (PE) ceramics and ferromagnetic (FM) alloys [6,7,8]. In applications, ME functional elements usually operate at electromechanical-resonance frequencies, where the ME coupling is enhanced due to the increased deformation amplitude [6,9,10,11,12]. Because of the potential applications of ME heterostructures as vibration energy harvesters and biomedical sensors, it would be desirable to reduce the resonance frequency (typically, 1–100 kHz in conventional composites) by three orders of magnitude. This can be achieved if the constitutive materials are soft ($Y\leq 10^{9}$ Pa). The lack of soft ME materials in nature stimulates the search for new concepts. Soft PE materials can be obtained from polymers, e.g., polyvinylidene fluoride (PVDF) [13]. A soft replacement for the rigid FM material in ME heterostructures is somewhat of a challenge [14]. Very recently, attention has been drawn to the soft (Y∼ 30–300 kPa), magnetoactive elastomers (MAEs) as promising candidates for the usage as magnetostrictive layers in layered multiferroic heterostructures [15,16]. MAEs consist of micrometer-sized ferromagnetic particles embedded in a soft polymer matrix. They exhibit exceptional magneto-mechanical effects [17,18,19,20]. In [21], helically coiled MAE stripes were used to realize untethered soft robots.

In an external magnetic field, a compliant MAE object may change its dimensions or shape (morph) [22]. If an applied magnetic field is homogeneous, the corresponding strain during the process of magnetization is often designated as magnetostrictive strain. A giant extensional strain up to ≈20% was recently observed in soft MAE cylinders [23], which makes the MAEs promising candidates for ME composites. MAEs can be combined with PE polymers in layered composite materials, where a significant direct ME effect is observable [24,25]. If a magnetic field is applied perpendicularly to the plane of the layered cantilever, the cantilever is bent due to the torque acting on the MAE layer [26,27], and the resulting deformation of the PE layer leads to the generation of an electric voltage. The proof of concept has been performed in inhomogeneous magnetic fields [16], which did not allow one to measure the ME voltage coefficient. The quasi-static ME voltage coefficient for the discussed type of ME heterostructures was later estimated to be about 50 V/A in a magnetic field of ≈100 kA/m [24]. The experiments in [24] were performed in uniform pulsed magnetic fields. In [16,24], the magnetic field was applied perpendicularly to the plane of the MAE samples. Such an arrangement is commonly designated as the T-T (transverse magnetic field and transverse electric field) configuration [1]. Very recently, a conventional L-T (longitudional magnetic field and transverse electric field) configuration was also investigated, where a superposition of constant and small alternating magnetic fields (standard method of sinusoidal field excitation [28]) was applied in the plane of the composite heterostructure [25]. The highest ME voltage coefficient of about 7.85 V/A was measured in a sample where the thickness of the MAE layer was ≈2 mm. The corresponding resonance frequency in the absence of a magnetic field was low ($f_{r}\approx 29$ Hz). The first results [16,24,25], obtained on multiferroic layered structures containing an MAE layer and a PE polymer, confirmed that these heterostructures are promising for the development of magnetic field sensors and energy harvesting devices. It seems that contrary to the conventional paradigm, the T-T configuration leads to a higher ME voltage coefficient than the L-T configuration. Mechanically soft, multiferroic cantilevers, such as those presented in this paper, can easily morph (bend to a great extent) towards the direction of an applied magnetic field so that their demagnetizing factor drastically decreases and the ME coupling becomes highly efficient [25]. Hitherto, multiferroic layered composites containing an MAE layer are not well investigated and not all the specific features of ME coupling in them are well understood, for example, the unexceptionally large increase in the resonance frequency in magnetic fields [25] or the retarded deflection and voltage response with respect to the magnetic field [24].

One specific feature which distinguishes the heterostructures in the present paper from conventional multiferroic laminates is the viscoelastic behavior of the magnetostrictive layer (MAE slab), which is field-dependent. As far as MAEs are concerned, it has been recently pointed out that special attention to deformation and magnetic rates is required [29]. However, even in the rapidly evolving field of MAEs, this aspect seems to be largely neglected.

The main goal of this paper is to investigate in detail the ME response of multiferroic heterostructures containing a PE polymer layer and an MAE slab in a triangle-wave magnetic field, in particular, the dependence on the magnetic field rate. Furthermore, we use the conventional method of harmonic (sinusoidal) magnetic field excitation at very low frequencies (≤3 Hz) and determine the optimum magnetic field for the effective ME coupling. In particular, a high ME voltage coefficient of ≈25 V/A is demonstrated at the excitation frequency of 0.1 Hz in the constant magnetic field of about 73 kA/m.

## 2. Materials and Methods

### 2.1. Multiferroic Cantilevers

**Figure 1:**
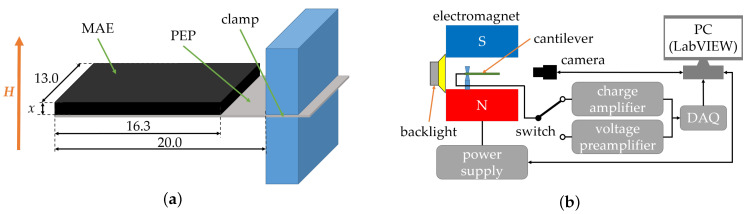
Sketch of a clamped cantilever [24] (**a**) and schematic of the experimental setup (**b**). Dimensions are given in mm.

Cantilevers comprised an MAE layer of varying thickness *x* and a commercially available PVDF-based vibration sensor (LDT0-028K, Measurement Specialties, Hampton, VA, USA) [30] (Figure 1a). For the sake of simplicity, the vibration sensor will be denoted in the following as a PE polymer (PEP). PEP was a flexible component comprising a thin (thickness $t_{PE}=28$ µm) PE PVDF polymer film with screen-printed silver ink electrodes, laminated to a 0.125 mm polyester substrate, and fitted with two crimped contacts. Because the PE film was displaced from the mechanical neutral axis, bending created a very high strain within the PVDF layer and high voltages were generated [31,32]. The fabrication procedure of heterostructures was described in detail in [24]. A thin, silicone-based adhesive layer, which is not sensitive to the magnetic field, was employed between the MAE layer and the vibration sensor. This layer highly increased the durability of bonding between different layers and enhanced the magnitude of ME coupling [24]. The MAE material contained 80 wt% (≈33 vol%) of carbonyl iron powder (CIP, type SQ, BASF SE, Ludwigshafen, Germany). The low-frequency shear storage modulus of the MAE material, obtained using a commercial rheometer (model Physica MCR 301, Anton Paar, Graz, Austria), was 50.4 kPa. This particular MAE material for composite cantilevers was selected because, in the previous work [24], it resulted in the highest voltage response in comparison to alternative compositions.

### 2.2. Experimental Setup

**Figure 2:**
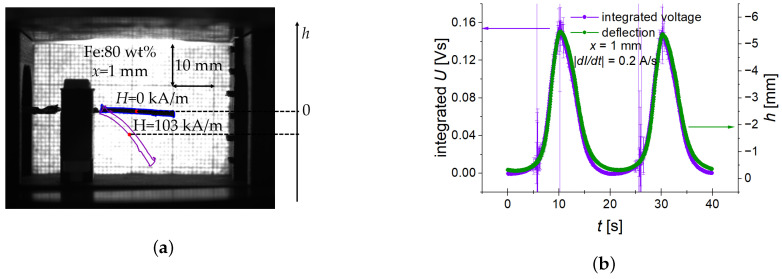
(**a**) Example of determining the deflection *h* from measurements of the cantilever’s “mass centre” [24]. (**b**) Comparison of the numerically integrated output voltage *U* (purple line) and the deflection *h* (green line) for the case of a slew rate of 12.2 kA/sm and x=1 mm.

A cantilever was clamped on the side of electrical contacts in a 3D printed holder from a polylactic acid (PLA) material (Figure 1a). It was placed horizontally between the electromagnet’s poles. A uniform magnetic field was applied vertically. The cantilever’s deflection was recorded using a camera (Alvium 1800 U-319m, Allied Vision Technologies GmbH, Stadtroda, Germany) with a suitable lens (Edmund Optic Double Gauss Focusable, 25 mm C-mount F4.0 1.3″, Barrington, NJ, USA). The backlight illumination employed light-emitting diodes (LED, Illuminant G4 Pen, Conrad Electronics, Hirschau, Germany) with a diffuser (Perspex diffuse, 2.5 mm, 3A Composites GmbH, Sins, Switzerland) (Figure 1b). A graph paper screen was placed between the LED and a cantilever. The graph paper is visible as the background in Figure 2a. The LED illumination had no significant effect on the temperature of a cantilever because of the absence of direct illumination and short measurement times less than ≈100 s. All experiments were performed under normal laboratory conditions at 22 ± 1 ${}^{{}^{\circ}}$C. The output electrical signal from the PE material was directed either to a voltage preamplifier (KISTLER 5165A, Winterthur, Switzerland) with an amplification coefficient k=1 and an input impedance of 10 MΩ or to a charge amplifier (KISTLER 5018, Winterthur, Switzerland), whose outputs were connected to a data acquisition (DAQ) board (NI USB-6212, National Instruments, Austin, TX, USA) linked to a personal computer (PC). The experiment was automated with LabVIEW software (National Instruments, Austin, TX, USA). By these means, either the generated voltage *U* or charge *Q* were measured as functions of time *t*.

### 2.3. Image Processing

The deflection of a cantilever was determined by image analysis using the OpenCV library in Python programming language. The deflection *h* was defined as the difference between the positions of the so-called “mass centre” which is the average of the vertical coordinate of the cantilever’s contour (Figure 2a). It is a measure of the cantilever’s deformation, which causes strain in the PE layer. We have found that the reference to the “mass centre” is a more robust procedure than the strategy to follow the tip of the cantilever. Furthermore, such a definition through the “mass centre” is meaningful because the generated voltage is practically proportional to the time derivative of the cantilever’s deflection [24]. An example is demonstrated in Figure 2b for the triangle-wave excitation and a magnetic slew rate of 12.2 kA/sm.

### 2.4. Measurement Protocol

The experimental setup was calibrated in such a way that the external magnetic field strength *H* was calculated from the electric current flowing through the electromagnetic coils. The resulting magnetic field strength was almost directly proportional to the excitation current. An alternating current had a symmetrical triangular waveform. However, there was a slight offset of not more than ≈2.4 kA/m, observed in the dependence of the magnetic field on the electric current due to some remanent magnetization of electromagnet’s pole shoes. The compensation of such a small offset was not possible due to the lack of necessary precision in setting the excitation current. The bi-polar power supply (FAST-PS 1k5, CAENels s.r.l., Basovizza, Italy) allowed one to adjust a constant rate for the rising or falling electrical current.

**Figure 3:**
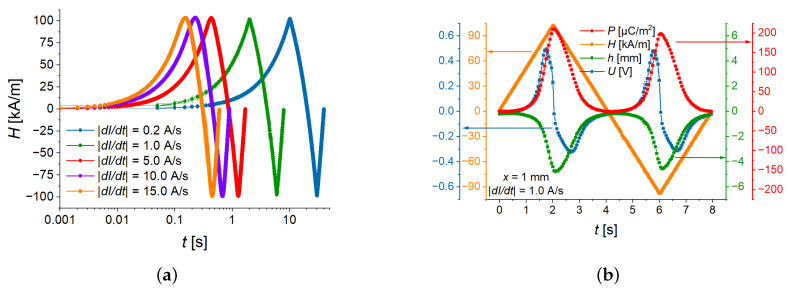
(**a**) One cycle of magnetic field oscillations in the logarithmic time scale for the case $H_{max}\approx 103$ kA/m. (**b**) Typical responses of polarization (red, *P*), deflection (green, *h*) and voltage (blue, *U*) to triangular waveform with a slew rate of 53 kA/sm (orange, *H*).

Five different slew rates for the electric current were used, as shown in Figure 3a and Table 1. The frequency *f* of the resulting magnetic field depended on the maximum electric current and the slew rate. The frequency in the last column of Table 1 was calculated for the maximum current $I_{max}=2.0$ A, which corresponds to the peak value of the magnetic field strength $H_{max}\approx 103$ kA/m.

Two sampling rates were used for measurements of physical quantities. The electric polarization or voltage were obtained at 1000 Hz, while the magnetic field was measured at 20 Hz. For the lower two slew rates in Table 1, the corresponding electrical quantities (charge, voltage) were taken at the same time points as the magnetic field and the deflection was measured. However, for the higher three rates, a cubic spline interpolation was used to interpolate from 20 Hz onto 1000 Hz.

Voltage and charge were monitored with a DAQ board. To remove some noise at mains frequency, a notch filter, centered at 50 Hz with a 3 dB bandwidth of 10 Hz, was applied to measured data. The electrical charge *Q* was re-calculated into the polarization as $P=\sigma {\left(1-\varepsilon_{r}^{-1}\right)}^{-1}$, where σ=Q⁄A, *A* is the effective area of the PE layer, $\varepsilon_{r}$ is the relative permittivity of the PE material, and a typical value $\varepsilon_{r}\approx 15$ was assumed [33].

A train of bi-polar current pulses comprising five periods of a symmetric triangular waveform was used at a fixed slew rate and an amplitude. The output signal (generated voltage or charge) and the applied magnetic field strength were recorded. Since MAEs exhibit hysteretic behavior of measured quantities with respect to the applied magnetic field [24,25,34,35], the response to each period of an applied magnetic field is somewhat different. A hysteresis in MAEs is commonly attributed to the magnetic-field-induced restructuring of filler particles, which is also the origin of magnetic hysteresis in such a material. The hysteresis of magnetic properties in MAEs, filled with soft-magnetic particles, was explained in [36] by the hysteresis of the consolidation of filler particles into elongated aggregates. However, it has been previously noticed that only an initial (magnetic field increase from zero to the peak value) curve significantly differs from the subsequent cycles [24,35]. A possible explanation is that during the first cycle major restructuring of the filler takes place, whereas further changes are minor [35]. To take into account the hysteretic behaviour of MAEs, the following approach was used for the evaluation of data. The results were presented over one period of the excitation current. The values at a particular time point within a period were averaged over the last four periods of the excitation current. The differences between the measured values at a particular time point in four subsequent periods are reflected by the error bars, with a confidence level of 95%.

The ME coupling coefficient $\alpha^{H}=\partial P/\partial H$ [28] at time point i·Δt was numerically calculated as $\left(P_{i}-P_{i-1}\right)/\left(H_{i}-H_{i-1}\right)$, where Δt is the time interval between measurement points and *i* is the consecutive number of a measurement point.

**Figure 4:**
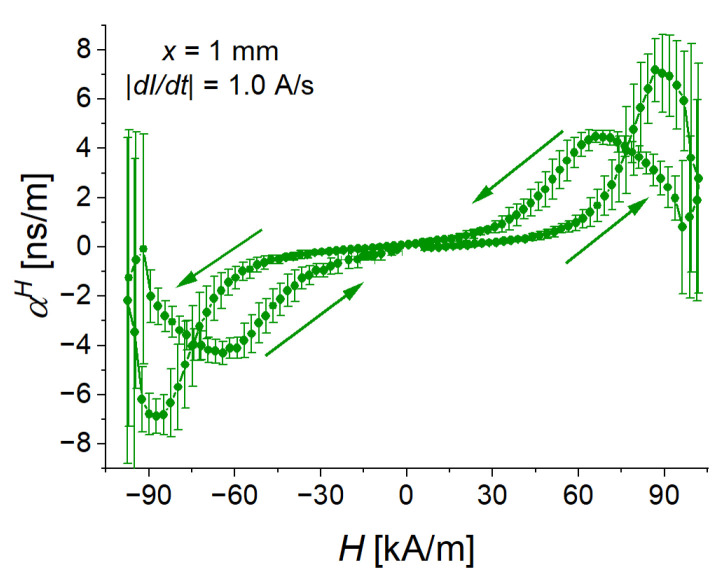
Dependence of the magnetoelectric coupling coefficient for x=1 mm and a slew rate of 53 kA/sm on the applied magnetic field strength. Here and in the following Figures, the arrows designate the direction of magnetic-field change.

As an example, Figure 4 presents the result of measurements of the ME coupling coefficient $\alpha^{H}$ for the triangular waveform with a slew rate of 53 kA/sm and a peak magnetic field strength of 103 kA/m. Here and in the following Figures, the field dependences are constructed by using the values at the same time moment. It is the only example of $\alpha^{H}$, where error bars will be shown. On all further graphs presenting the ME coupling coefficient $\alpha^{H}$, error bars are not shown. These error bars may become too large due to the use of a numerical derivative of raw data, which is sensitive to the presence of some noise. This is typically the case in the vicinity of the local maxima and minima of the magnetic field, where its time derivative changes its sign. In the following, an additional running average was run over ME coupling coefficient data to smoothen the first derivative. The moving average was run over five points for the lower two rates in Table 1 and over fifteen points for the higher three rates in Table 1.

## 3. Results

### 3.1. Effect of Magnetic-Field Slew Rate

**Figure 5:**
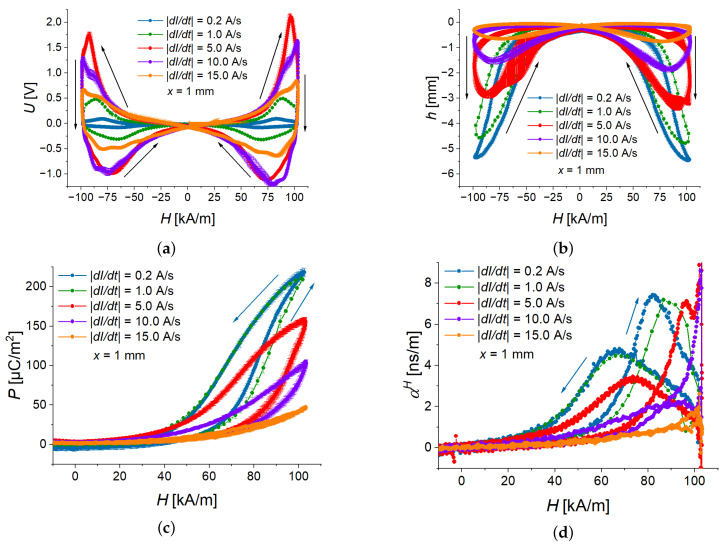
Average field dependences of generated voltage (**a**), deflection (**b**), polarization (**c**), and ME coupling coefficient (**d**) for different slew rates.

Five different slew rates were investigated for a sample with the MAE thickness x=1 mm. In Figure 5, you can see the field dependences of measured physical quantities. The generated voltage is shown in Figure 5a, where one can notice come difference between peak values in positive and negative magnetic fields. This difference is caused by a slight remanent magnetization of the electromagnet’s shoe poles and it is clearly visible only in the voltage measurement. The highest ME voltage was achieved at an intermediate slew rate of 254 kA/sm.

As all measured physical quantities are almost perfectly symmetric either with respect to the *y* axis (voltage, deflection, polarization) or with respect to the origin of the graph (ME coupling coefficient), Figure 5c,d and the following Figures show only the results for the positive values of the applied magnetic field.

**Figure 6:**
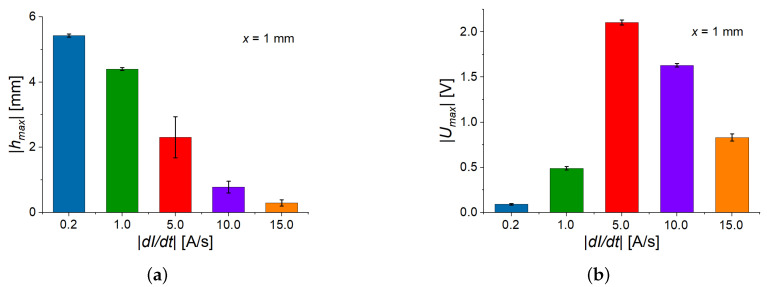
Peak deflection (**a**) and peak induced voltage (**b**) for different slew rates.

In Figure 5b and Figure 6a, it is seen that the deflection amplitude decreases with the increasing rate of the magnetic field. This can be explained by the delayed magneto-mechanical response of the viscoelastic material. With increasing slew rate, it becomes increasingly difficult for the cantilever to follow the magnetic field. Because the time derivative of the cantilever’s deflection *h* can be written as $dh/dt=\left(dh/dH\right)\cdot \left(dH/dt\right)$, the interplay between the delayed magneto-mechanical response (at fixed external magnetic field the derivative dh⁄dH decreases with increasing magnetic rate) and the increase in the magnetic rate (dH⁄dt) leads to the appearance of the highest peak value $|U_{max}|$ of the generated voltage U(t) at an intermediate value of |dH⁄dt| (Figure 6b). Note that the deflection has negative values due to the selected direction of the coordinate axis (Figure 2). How the direction of deflection can be influenced was discussed in [24].

**Figure 7:**
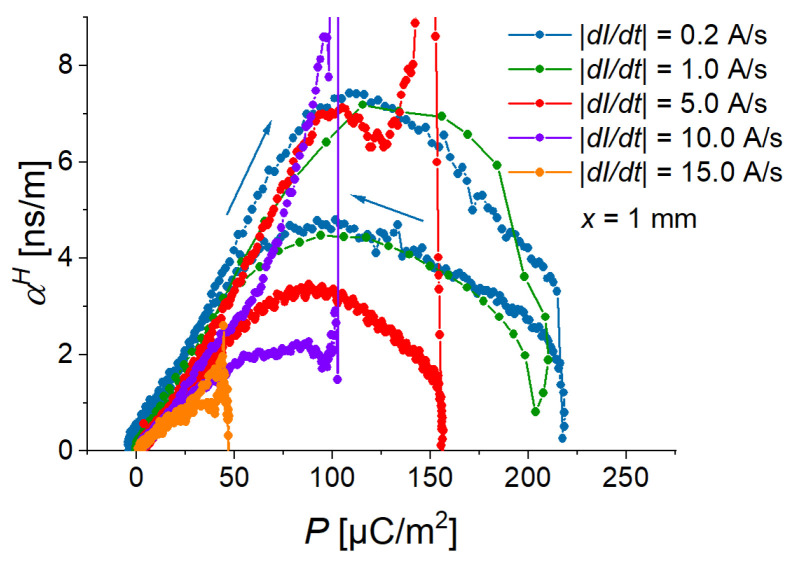
Dependences of the ME coupling coefficient on the electric polarization for different slew rates.

Figure 5c presents the field dependence of the (averaged) polarization. It can be observed that its field behavior is similar to the field behavior of the cantilever’s deflection. Figure 5d compares the field dependences of the ME coupling coefficient at different magnetic-field rates. The magnitude of a ME coupling coefficient has two local maxima—one (“advancing”) maximum for the ascending branch of the magnetic field magnitude and another (“receding”) maximum for the descending branch of the magnetic field magnitude. Advancing maximum is higher and narrower than receding maximum. Both maxima of $\alpha^{H}\left(H\right)$ “shift” to the higher values of magnetic field with increasing slew rate. For the two fastest rates (10 A/s, 15 A/s), the local maxima in the field dependences of $\alpha^{H}$ are not observable in the measurement range of the magnetic field. Remarkably, the local maxima of $\alpha^{H}$ correspond to particular values of polarization *P*: the maximum value of $\alpha^{H}$ for the ascending magnetic field occurs at P=(110±13) µC/m${}^{2}$, while the maximum value of $\alpha^{H}$ for the descending magnetic field occurs at P=(97±10) µC/m${}^{2}$, independently of the magnetic rate (Figure 7). We believe that this observation should be related to the ferroelectric hysteresis loop of the PE PVDF film and this phenomenon should be investigated in detail in future works.

### 3.2. Effect of MAE Thickness

**Figure 8:**
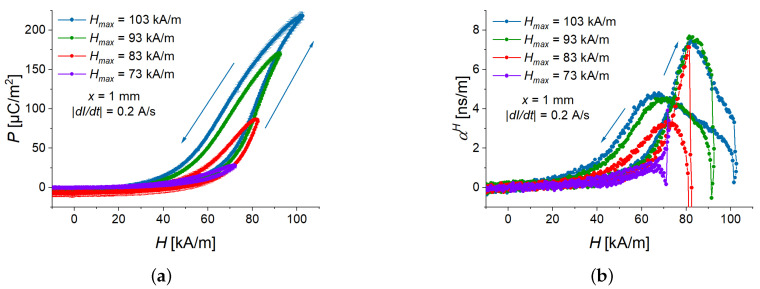
Field dependences of the electric polarization (**a**) and the ME coupling coefficient (**b**) for different thicknesses of MAE layer. The magnetic field slew rate is 12.2 kA/sm.

Next, we investigated the effect of MAE thickness on the ME response. Four cantilevers with different MAE thicknesses *x* were compared at a fixed slew rate of 12.2 kA/sm. As reported in [24], in a fixed magnetic field, the deflection increases with the increasing thickness of the MAE layer. The reason is that the torque in a magnetic field increases with increasing amount of magnetic material (total mass of iron particles). Since the maximum possible deflection of a cantilever (h≤6 mm) is limited by the geometry of the experimental setup, the amplitude of the magnetic field was adjusted according to the thickness of the MAE layer (Table 2). Obviously, this results in different frequencies of the applied magnetic field. Figure 8 depicts the results of measurement of the field dependences of the electrical polarization and the ME coupling coefficient.

**Figure 9:**
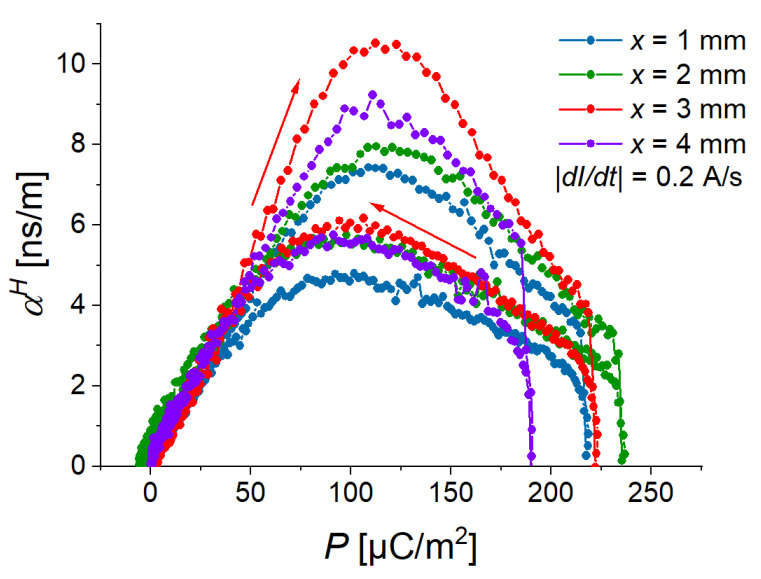
Dependences of ME coupling coefficient on electric polarization for different thicknesses of MAE layer and the magnetic slew rate of 12.2 kA/sm.

The highest “advancing” maximum of the ME coupling coefficient was observed for a cantilever with an MAE thickness of 3 mm. At a fixed magnetic field rate and a fixed deflection amplitude, there seems to be an optimum MAE thickness for the maximal ME coupling. Similar to what is observed in Section 3.1, the local maxima of $\alpha^{H}$ correspond to particular values of polarization *P*: the maximum value of $\alpha^{H}$ for the ascending magnetic field occurs at P=(114±10) µC/m${}^{2}$, while the maximum value of $\alpha^{H}$ for the descending magnetic field occurs at P=(99±10) µC/m${}^{2}$, independently of the MAE thickness (Figure 9).

### 3.3. Effect of Magnetic-Field Amplitude

**Figure 10:**
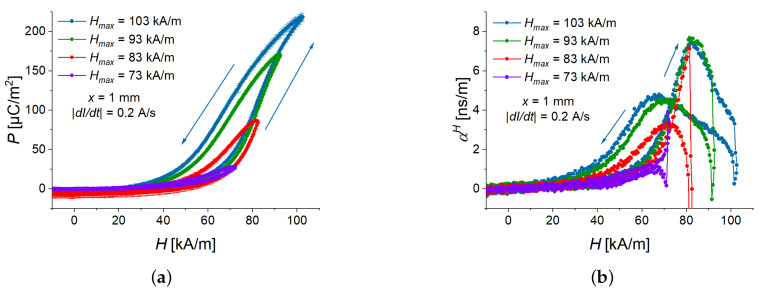
Field dependences of polarization (**a**) and ME coupling coefficient (**b**) for four peak values of magnetic field ($H_{max}=73$, 83, 93, and 103 kA/m) for a sample with 1 mm thick MAE layer.

**Figure 11:**
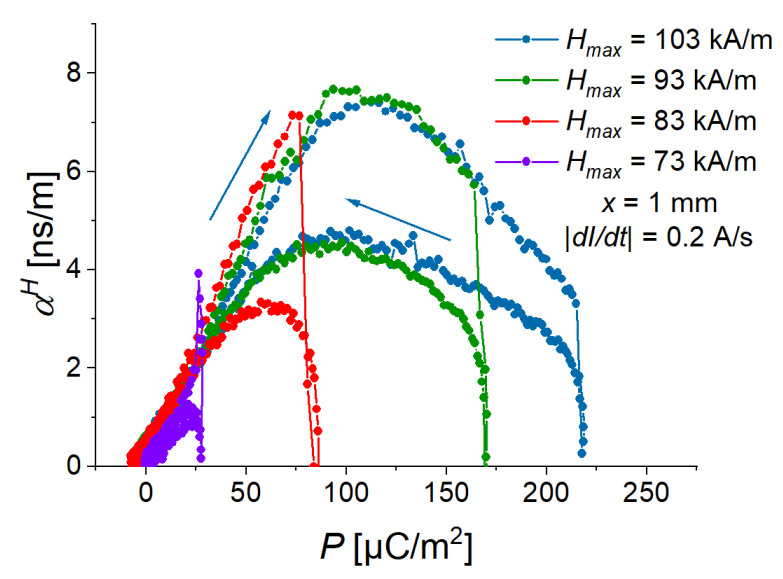
Dependence of the ME coupling coefficient on the electric polarization for four peak values of magnetic field ($H_{max}=73$, 83, 93, and 103 kA/m) and a sample with an MAE layer thickness of 1 mm at the magnetic slew rate of 12.2 kA/sm.

How does the amplitude of magnetic field affect the polarization and the ME coupling coefficient? The measurements were performed for a cantilever with an MAE thickness of 1 mm at a fixed magnetic field rate of 12.2 kA/sm. Figure 10 presents the measurement results. From Figure 10a, it is seen that the field dependence of the polarization for the ascending magnetic field remains the same (the curves practically overlap), while the polarization for the descending magnetic field is significantly different due to the effect of hysteresis. A similar effect is observed in Figure 10b for the ME coupling coefficient. Figure 11 shows the dependences of the ME coupling coefficient on the electric polarization. For the two smallest amplitudes of the triangle-wave magnetic field (73 kA/m, 83 kA/m), the local maximum of $\alpha^{H}\left(H\right)$ is not observed for the ascending magnetic field, because the optimum value of polarization of ≈110 µC/m${}^{2}$ is not reached. For these two magnetic-field amplitudes, the local maximum of $\alpha^{H}\left(H\right)$ for the descending magnetic field is an artifact, related to the numerical differentiation needed to calculate $\alpha^{H}\left(H\right)$. The optimum polarization value of ≈100 µC/m${}^{2}$ for the descending magnetic field is not reached. The highest ME coupling coefficient of ≈7.5 ns/m is obtained in the ascending magnetic field for the two highest peak values of magnetic field (93 kA/m, 103 kA/m) at P≈110 µC/m${}^{2}$.

### 3.4. Comparison of Triangular and Sinusoidal Waveforms

**Figure 12:**
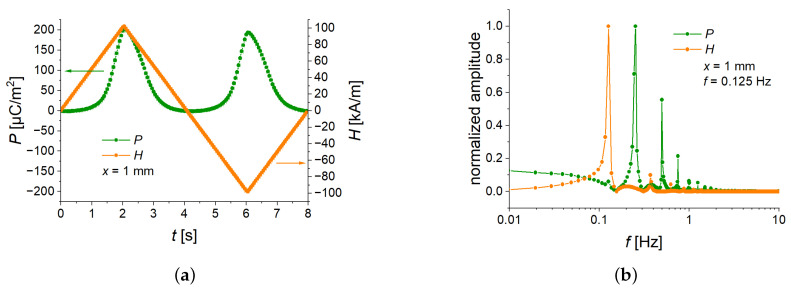

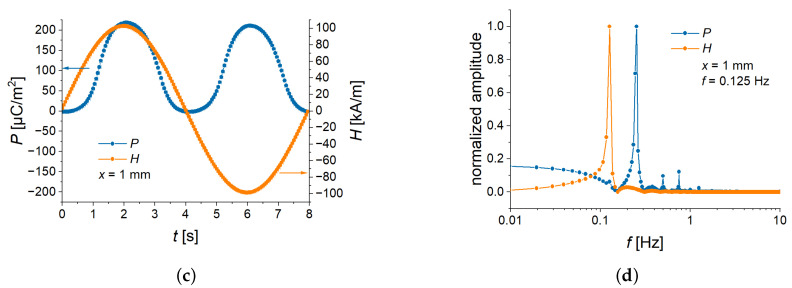
One average pulse of magnetic field and time dependence of the polarization response for symmetric triangle-wave (**a**) and sine-wave (**c**) magnetic field. FFT of magnetic field and electric polarization for symmetric triangular (**b**) and sinusoidal (**d**) waveforms of magnetic field excitation. The amplitudes of the FFT spectra are normalized to their maximum values.

**Figure 13:**
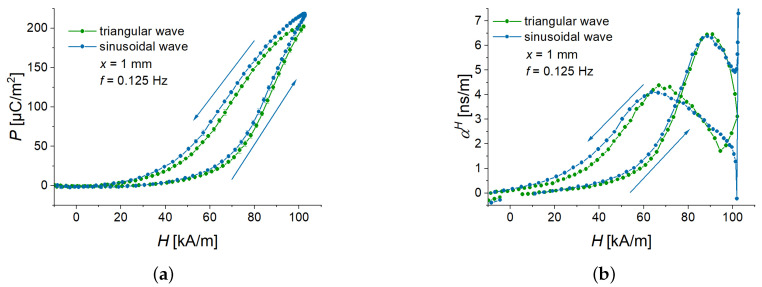
Comparison of field dependences of the polarization (**a**) and the ME coupling coefficient (**b**) for two different magnetic-field excitations. Symmetric triangular and sinusoidal waves have a frequency of 0.125 Hz.

To investigate the effects of magnetic-field slew rate, the triangular waveform is an obvious choice. However, conventional experimental characterizations involve sinusoidal wavefunction. To investigate the difference between triangular and sinusoidal magnetic fields, we used a feature of the available power source. To obtain comparable data, a point-by-point protocol was created with a sampling rate of 20 Hz. The slew rate of the power supply was set to the highest possible value of 15 A/s (757 kA/sm). The current was measured at 15 ms after it was set. The period duration of the sinusoidal voltage was 8 s (f=0.125 Hz), this corresponded to 160 measurement points per cycle. For the fast Fourier transform (FFT) analysis, 12 cycles were used. The data in Figure 12 and Figure 13 are the averaged values over the last 11 cycles, from which also the uncertainties of measurements have been obtained.

Figure 12 presents the time behavior of the averaged polarization for one cycle of applied magnetic fields. As was expected, this behavior was different for triangular and sinusoidal waveforms. The polarization had a double repetition frequency with respect to the applied magnetic field (Figure 12a,c) because the cantilever always deflects in the same direction, independently of the polarity of an applied magnetic field. The corresponding spectra (Figure 12b,d) demonstrated the presence of the double frequencies of the excitation field. Figure 12d clearly shows that our synthesis of a sinusoidal field was successful because there was one dominating frequency in the FFT spectrum. As expected, the Fourier spectrum of the symmetric triangle-wave function also had higher harmonics [37,38].

However, Figure 13 demonstrates that the field dependences of the measured polarization and calculated ME coupling are very similar. Therefore, the results presented in this paper can be reasonably applied to sinusoidal magnetic fields with the same frequency and amplitude if field dependences are considered.

## 4. Discussion

The above results clearly demonstrate that the ME response of multiferroic cantilevers containing an MAE layer depends on the slew rate and the amplitude of a magnetic field strength. We attribute this fact to the viscoelastic nature of MAE materials as it was pointed out in [29].

Furthermore, as already reported in [24], measured physical quantities (voltage, deflection, polarization) and calculated ME coupling coefficient have hysteresis behavior with respect to the applied magnetic field.

At a particular momentary value of the calculated external magnetic field, most of the measured physical quantities (deflection, polarization) and ME coupling coefficient did not reach their steady-state (maximum) value, which would be possible for the step magnetic field excitation because the magneto-mechanical response is retarded with respect to the magnetic field. Obviously, this is also the origin of observed hysteresis behavior. As far as the measured voltage is concerned, under a step field excitation, it vanishes in the steady-state due to the finite electrical impedance of the used pre-amplifier.

**Figure 14:**
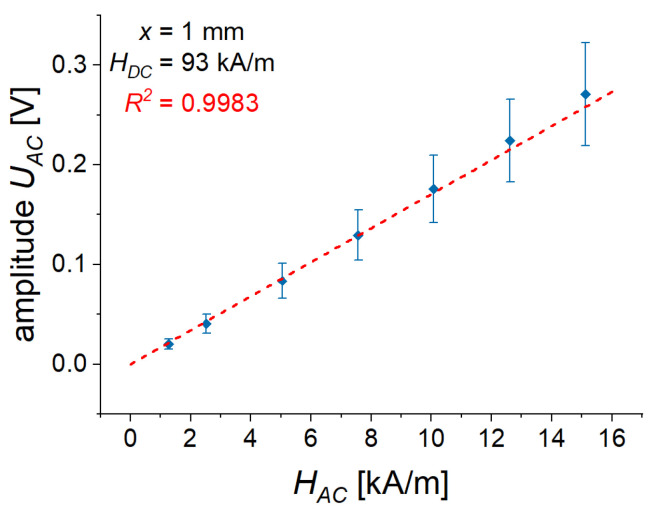
Dependence of the AC voltage amplitude $U_{AC}$ on the amplitude $H_{AC}$ of AC magnetic field. $R^{2}$ denotes the determination coefficient of the linear regression.

In the above considerations, alternating magnetic fields were employed. How are the determined values of the ME coupling coefficient compared with those obtained by a conventional method of harmonic field modulation (HFM) [28]? To answer this question, an AC magnetic field with an amplitude $H_{AC}$ of 5 kA/m, which was in the linear region of voltage response $U(H_{AC})$ (Figure 14), was superimposed on a constant (bias) magnetic field $H_{DC}$. Note that $H_{AC}\ll H_{DC}$.

(1) $$H\left(t\right)=H_{DC}+H_{AC}\cos\left(2\pi ft+\varphi_{H}\right),$$

(2) $$P\left(t\right)=P_{slow}\left(t\right)+P_{AC}\cos\left(2\pi ft+\varphi_{P}\right).$$

P(t) is the time dependence of the polarization *P*, $P_{AC}$ is the amplitude of the sinusoidally varying component of the polarization, $P_{slow}\left(t\right)$ is the transient component of polarization, which will be explained below. $\varphi_{H}$ and $\varphi_{P}$ are the initial phases of magnetic field and polarization, respectively.

The essential difference between the above experiments is that the constant field $H_{DC}$ was not zero. Therefore, the generated electric charge oscillated at the excitation frequency and not at the double excitation frequency as in the experiments above. $H_{DC}$ varied between 53 and 108 kA/m with a step of 5 kA/m. The value of $H_{AC}$ was equal to the step size. The frequency value *f* were 0.1, 0.5, 1.0, 2.0, and 3.0 Hz. The frequency f=3.0 Hz was maximal possible due to the time discretization of the excitation current and the time resolution of our measurement setup (20 Hz). At this frequency, the applied AC magnetic field still had a reasonable sinusoidal waveform (7 points per cycle).

As an example, Figure 14 demonstrates that the amplitude $H_{AC}$ of the sinusoidal magnetic field was small enough, and that the linear ME effect was investigated.

**Figure 15:**
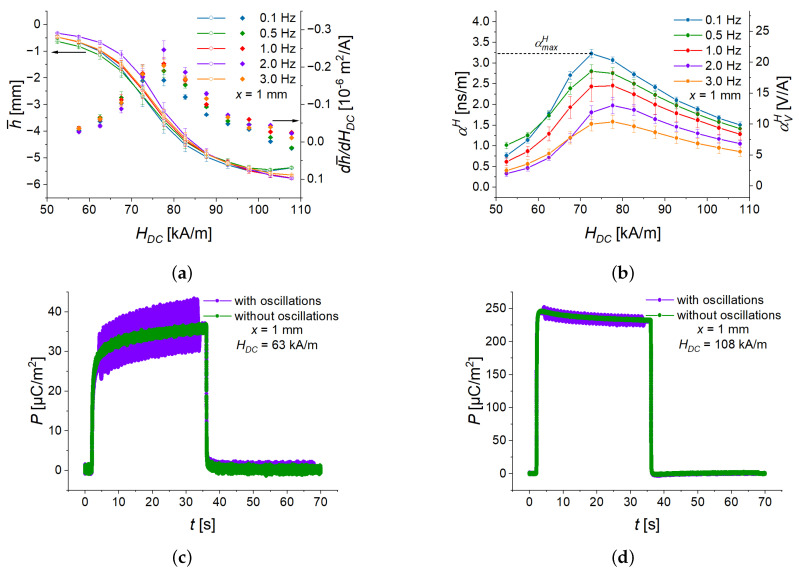
(**a**) Field dependences of the deflection and its derivative with respect to the constant magnetic field. (**b**) Field dependences of the ME coupling coefficient for five different frequencies. Examples of the transient behaviour of polarization for the excitation current of 1.2 A (**c**) and 2.1 A (**d**). As an example, $\alpha_{max}^{H}$ shows the maximum value of $\alpha^{H}$ for f=0.1 Hz.

Similar to the measurements above, charge, magnetic field, and deflection were acquired. The average deflection for different frequencies and magnetic fields is shown in Figure 15a. The electric charge was re-calculated into the polarization. The transient component $P_{slow}\left(t\right)$ of P(t) was subtracted from the signal, so that only the sinusoidal function remained. The examples of the transient behaviors are shown in Figure 15c,d. Finally, the sinusoidal functions were fitted to the experimentally obtained magnetic field and polarization according to Equations (1) and (2). From the obtained amplitudes of fits, the ME coupling coefficient was calculated as:

(3) $$\alpha^{H}=\frac{P_{AC}}{H_{AC}}.$$

The obtained ME coupling coefficient is shown in Figure 15b. At the lowest frequency, the ME coupling coefficient reaches 3.2 ns/m (the ME voltage coefficient ≈ 25 V/A). Similarly to the electric polarization, the cantilever’s deflection oscillates at a given frequency. Therefore we introduced the deflection $\bar{h}$, which is the mean value of deflection over several oscillation cycles. Figure 15a also shows numerically calculated derivatives of the averaged deflection $\bar{h}$ with respect to the applied DC magnetic field $d\bar{h}⁄dH_{DC}$. The maximum of this derivative is always observed at $H_{DC}\approx 73$ kA/m. The derivative $d\bar{h}⁄dH_{DC}$ can be seen as an analog of the piezomagnetic coefficient in the conventional theory of the ME effect in composite multiferroic materials. It is visible from Figure 15b, that the maximum of the ME coupling coefficient is observed at approximately the same value of the constant magnetic field as for the derivative $d\bar{h}⁄dH_{DC}$. The mechanism of the optimum low-frequency ME coupling seems to be similar to conventional ME multiferroic layered composites. We also recalculated the ME coupling coefficient $\alpha^{H}$ into the ME voltage coefficient $\alpha_{V}^{H}=\alpha^{H}{\left(\varepsilon_{0}\varepsilon_{r}\right)}^{-1}$[28,39], where $\varepsilon_{r}$ is the relative permittivity of the PE material, and a typical value $\varepsilon_{r}=15$ was assumed [33]. The ME voltage coefficient can be easily expressed in CGS units, taking into account that 1 Oe corresponds to 79.577 A/m.

We also observed the characteristic behaviour of the transient component $P_{slow}\left(t\right)$ changes around the magnetic field value of $H_{DC}=88$ kA/m. Below this value, the polarization $P_{slow}\left(t\right)$ reaches the steady-state exponentially as $P_{slow}\left(t\right)=P_{max}\left(1-e^{-kt}\right)$ (Figure 15c). Above $H_{DC}=88$ kA/m, first an overshoot was observed and then $P_{slow}\left(t\right)$ relaxed exponentially as $P_{slow}\left(t\right)=P_{0}e^{-kt}$ (Figure 15d). Qualitatively similar changes in the transient behavior of the magnetodielectric effect (magnetic-field-dependent permittivity) [35] and the engineering stress [29] were previously observed. In [29], four different magnetic rates were compared at constant step magnitude. At lower magnetic rates (2 and 20 mT/s), the behavior of engineering stress was qualitatively similar to the transient behavior in Figure 15c. For the higher two magnetic rates (200 and 1000 mT/s), the transient behavior of stress was similar to that in Figure 15d. When applying a magnetic field with a slow slew rate, the particular transient behavior was attributed to the balance between magnetic forces between magnetized particles and the viscoelasticity of the polymer matrix. For the higher two slew rates, an alternative explanation was suggested. In this case, the behavior was assigned to the microstructural stiffening of polymeric matrix, which results in a “collapse” of magnetic particles being unable to properly rearrange along magnetic field lines, leading to a microstructural blockade [29].

**Figure 16:**
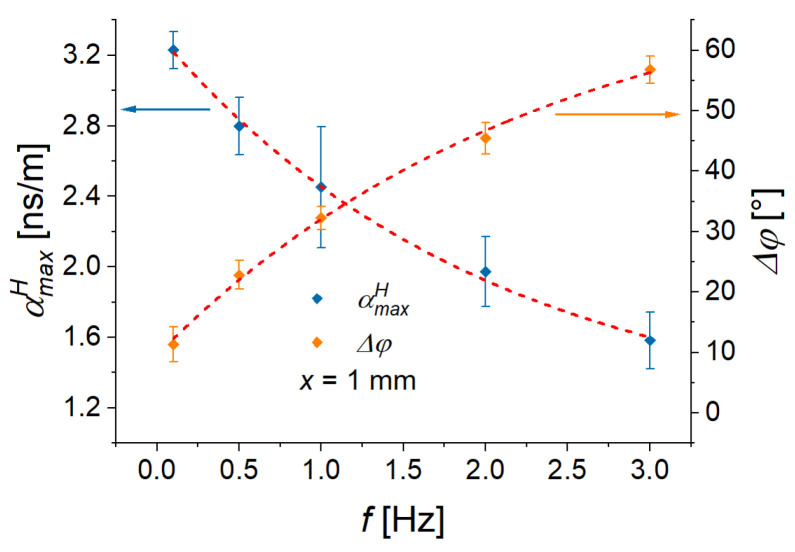
Frequency dependences of the peak value of the ME coupling coefficient $\alpha_{max}^{H}$ and the phase shift Δφ. Dashed lines indicate fitted exponential functions ($y\left(f\right)=a+be^{-kf}$), where *y* is either $\alpha_{max}^{H}$ or Δφ.

Figure 16 presents the frequency dependencies of the maximum ME coupling coefficient $\alpha_{max}^{H}$, extracted from Figure 15a and the phase difference $\Delta \varphi =\varphi_{H}-\varphi_{P}$ between the alternating magnetic field and the alternating component of polarization. The maximum ME coupling coefficient increases with the decreasing excitation frequency. The reason for that can be the decreasing phase delay between the magnetic field and the polarization so that the ME coupling becomes more efficient. The data shown in Figure 16 have been fitted to the function $y\left(f\right)=a+be^{-kf}$. The results of fitting are summarized in Table 3.

From the exponential fit, the quasi-static (f→0 Hz) phase difference Δφ of $9.6^{{}^{\circ}}\pm 13.5^{{}^{\circ}}$ can be estimated. Therefore, it can be reasonably assumed that in a quasi-static state, the ME response can follow the applied magnetic field ($\Delta \varphi \to 0^{{}^{\circ}}$). The phase delay of the polarization increases with increasing frequency and tends asymptotically to roughly 74.24°, if higher resonance frequencies are neglected. Similarly, the quasi-static ME coupling coefficient can be estimated to be ≈3.3 ns/m (ME voltage coefficient ≈25.1 V/A). One can conclude that the low-frequency ME voltage coefficient, obtained using the conventional method of sinusoidal magnetic-field excitation [28], has the same order of magnitude as previously reported value from magnetic-field excitations [24]. The maximum obtained value of the low-frequency ME voltage coefficient is more than three-fold higher than the largest ME voltage coefficient obtained at a resonance frequency on similar structures in the L-T geometry [25]. Note that the results presented at very low excitation frequencies (≤3 Hz); the multiferroic heterostructures were not operating at resonance. It can be expected that the ME voltage coefficient can be further enhanced at higher frequencies, where electromechanical resonances can be observed [25].

We are aware of only a few papers, where ME coefficients at such low frequencies have been reported. The measured maximum value of $\alpha_{V}^{H}$ is two orders of magnitude higher than that obtained [40] at ≈1 Hz utilizing the concept of the so-called magnetoelectric electret. In [41], the reported ME coupling coefficient of a heterostructure composed of six layers of a magnetostrictive amorphous alloy (Metglas^®^, $Y∼10^{11}$ Pa) and a piezoelectric composite core of five PMN-PT fibers, interrogated by a pair of Kapton inter-digited (ID) electrodes, was $\alpha_{Q}^{H}\approx 34$ ps·m at f=1 Hz, which is about two orders of magnitude higher than measured here ($\alpha_{Q}^{H}\approx 0.35$ ps·m). Several interesting experiments were performed [42] at low frequencies, but the ME coefficient was not reported.

**Figure 17:**
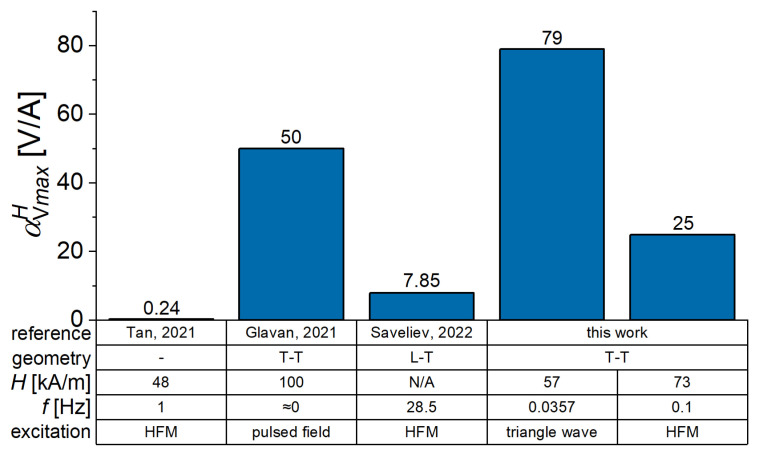
Comparison of different reported values of the ME coupling coefficient at low frequencies. (N/A stands for not available; Tan, 2021 stands for [40]; Glavan, 2021 stands for [24] and Saveliev, 2022 stands for [25]).

Finally, Figure 17 compares the highest values of ME voltage coefficient obtained with MAE/PEP heterostructures for different magnetic field excitations. It is seen that investigated structures compare well with an alternative technology [40].

## 5. Conclusions

We have investigated the direct ME effect in multiferroic cantilevers containing an MAE layer in the alternating triangle-wave magnetic field. Furthermore, we measured the ME voltage coupling coefficient in the T-T geometry using sinusoidal magnetic-field excitation at low frequencies (f≤3 Hz). The following main conclusions can be made:

- The interplay between the delayed magneto-mechanical response and the increase in the magnetic slew rate led to the highest generated voltage at an intermediate value of the magnetic slew rate of ≈254 kA/sm;
- Two local maxima in the field dependence of the ME coupling coefficient were observed. One maximum appeared at ascending (increasing) magnetic field, the other maximum appeared at descending (decreasing) magnetic field. The maxima corresponded to particular polarization values: P≈110 µC/m${}^{2}$ for the ascending magnetic field and P≈100 µC/m${}^{2}$ for the descending magnetic field. Polarization values, where maxima of $\alpha^{H}$ were located, varied neither with the magnetic field slew rate nor with the thickness of the MAE layer;
- Field dependences of ME responses to triangular and sinusoidal waveforms of applied magnetic field were very similar. The electric polarization oscillated at double excitation frequency because the cantilever’s magnetomechanical response did not distinguish between different polarities of the magnetic field;
- Systematic measurements of the ME coupling coefficient at sinusoidal magnetic-field modulation were performed at very low frequencies (up to 3 Hz). The highest ME voltage coefficient was observed in the constant magnetic field $H_{DC}\approx 73$ kA/m. From these results, it was estimated that the highest ME coupling coefficient $\alpha^{H}$ at f→0 Hz is ≈3.3 ns/m (ME voltage coefficient ≈25 V/A or ≈20 Vcm${}^{-1}$Oe${}^{-1}$ in CGS units);
- Due to the viscoelastic properties of the MAE material, a phase delay between the applied magnetic field and electric polarisation was observed for sinusoidal magnetic-field excitations in the presence of a constant magnetic field. The phase delay increased with the increasing frequency *f*.

Further research is required to better understand and further enhance the ME coupling in the presented heterostructures. In particular, the increase in the excitation frequency of sinusoidal magnetic-field modulation may lead to a significant increase in the ME coupling in the T-T geometry due to the resonance enhancement.

## Figures and Tables

**Table 1:** Slew rates and frequencies of the magnetic field for cases shown in Figure 3a, where the maximal field amplitude was $H_{max}\approx 103$ kA/m.

$\left|\frac{\mathrm{dI}}{\mathrm{dt}}\right|$ [A/s]	$\left|\frac{\mathrm{dH}}{\mathrm{dt}}\right|$ [kA/sm]	$f\left(H_{\max}=103\;\mathrm{kA}/m\right)$ [Hz]
0.2	12.2	0.025
1.0	52.5	0.125
5.0	253.7	0.625
10.0	505.3	1.250
15.0	756.9	1.875

**Table 2:** Amplitudes of triangle-wave magnetic fields and their corresponding frequencies for different thicknesses of MAE layer at the magnetic slew rate of 12.2 kA/sm.

***x* [mm]**	1	2	3	4
$H_{\max}$ **[kA/m]**	103	83	73	63
***f* [mHz]**	25.0	31.3	35.7	41.7

**Table 3:** Parameters of fitted functions in Figure 16. $R^{2}$ denotes the determination coefficient.

Parameter	$\alpha_{\max}^{H}$	Δφ
*a*	1.10±0.22	74.24±7.22
*b*	2.23±0.20	−64.69±6.33
*k*	0.50±0.10	0.43±0.09
$R^{2}$	0.998	0.997

## Data Availability

The data that support the findings of this study are available from the corresponding author, G.G., upon reasonable request.
